# Importance of the Hounsfield Unit Value Measured by Computed Tomography in the Differentiation of Hydronephrosis and Pyonephrosis

**DOI:** 10.7759/cureus.11675

**Published:** 2020-11-24

**Authors:** Abdullah Erdogan, Murat Sambel, Volkan Caglayan, Sinan Avci

**Affiliations:** 1 Urology, Erzincan Binali Yildirim University Faculty of Medicine, Erzincan, TUR; 2 Urology, Bursa City Hospital, Bursa, TUR

**Keywords:** hounsfield unit, hydronephrosis, non-contrast-enhanced ct, percutaneous nephrostomy, pyonephrosis

## Abstract

Objectives

To evaluate the efficacy of the non-contrast-enhanced computed tomography (NCECT) renal pelvis Hounsfield unit (HU) values in differentiating between the hydronephrosis and pyonephrosis in dilated urinary systems.

Materials and methods

Patients who underwent percutaneous nephrostomy (PN) due to urinary system obstruction in the last three years were retrospectively evaluated. Pyonephrosis and hydronephrosis groups were differentiated according to the clarity of percutaneous needle aspiration. The patients’ renal pelvic anteroposterior (AP) diameter, renal pelvic area, and mean HU values were measured on NCECT and compared between two groups.

Results

PN was performed on a total of 523 patients. The study included 159 patients and 214 renal units. Hydronephrosis was detected in 176 renal units and pyonephrosis in 38 renal units. No statistically significant difference was observed between the measured AP diameter and renal pelvic area in the two groups (28.45 ± 10.1 mm vs. 31.13 ± 14.4 mm, p = 0.36 and 658.51 ± 433.1 mm^2^ vs. 755.14 ± 470.6 mm^2^, p = 0.22, respectively). The mean HU value of the pyonephrosis group was significantly higher (2.30 ± 5.02 vs. 10.97 ± 6.68, p < 0.001). At the cut-off value of 8.46, HU had a sensitivity of 68.4% and specificity of 92.6% in the diagnosis of pyonephrosis.

Conclusions

It is possible to determine differential diagnosis between pyonephrosis and hydronephrosis easily and without additional cost by performing dilated renal pelvis HU measurements on NCECT.

## Introduction

Acute or chronic obstruction of the urinary system can be caused by congenital anomalies, urolithiasis, urogenital tumors, and stenosis, as well as non-urologic malignancies. If pyonephrosis is present in acute obstruction, patients present with the triad of side pain, fever, and elevated white blood cell (WBC) count, and also labile hypotension can be seen depending on the severity of the disease. Patients who are not treated with appropriate decompression may develop septic shock. It was reported that 85% of patients with urosepsis and associated septic shock had an underlying urinary tract obstruction [1-3]. As well as elucidating the etiology of the obstructed urinary system, the differentiation between hydronephrosis and pyonephrosis is also important for determining the urgency of the need for treatment. Although the diagnosis of pyonephrosis is based on clinical, laboratory, and radiological findings, it may not be possible to differentiate it from hydronephrosis due to similar findings on ultrasonography [4]. In addition, if there is complete obstruction, the urethral urine sample may not reflect the current state of the upper urinary tract [5]. When obstruction is bilateral or if the patient with a solitary kidney develops obstruction, urgent decompression is required in the treatment of cases presenting with acute renal failure (ARF). Percutaneous nephrostomy (PN) has been used effectively and safely since it was first described in 1955 for the treatment of both pyonephrosis and bilateral obstruction [6].

Not only is non-contrast-enhanced computed tomography (NCECT) an easy- and fast-to-perform method for elucidating the etiology of obstruction, but it may also provide useful diagnostic information [7,8]. Using CT, the attenuation of tissues, body cavities, and fluids can be measured quantitatively and in a standard manner in the Hounsfield unit (HU) [9]. There are studies in the literature on the HU of the content of fluids accumulated in body cavities [10-12]. Yuruk et al. reported that in case of urinary tract obstruction, renal pelvis HU values measured on NCECT may be useful in differentiating between hydronephrosis and pyonephrosis [13]. In our study, we examined patients who underwent PN in our clinic due to urinary tract obstruction and investigated the efficacy of the pre-procedure non-contrast-enhanced renal pelvis HU value in differentiating between the two conditions.

## Materials and methods

After obtaining the approval of the ethics committee of the university, 523 patients who underwent PN due to urinary system obstruction between January 1, 2017, and October 1, 2019, were retrospectively reviewed. This study included 159 cases that underwent NCECT up to 24 hours prior to the procedure, underwent pyonephrosis-hydronephrosis differentiation using percutaneous needle aspiration during the procedure, and were detected to have the aspirated culture results. The patients who underwent bilateral PN, whose aspiration side and urine culture result were not clearly stated, and if the aspirated material was hemorrhagic were excluded from the study. All patients were intravenously administered 1 g ceftriaxone for prophylaxis 30 minutes before the procedure. The PN procedure was performed under local anesthesia by urologists. After the first needle entry was performed by ultrasonography in the prone position, the opaque substance was injected into the system. Following the confirmation of the location of the needle under fluoroscopic guidance, adequate dilatation was performed under fluoroscopy. The first aspirated sample obtained from the calyceal system was sent for a culture analysis.

**Figure 1:**
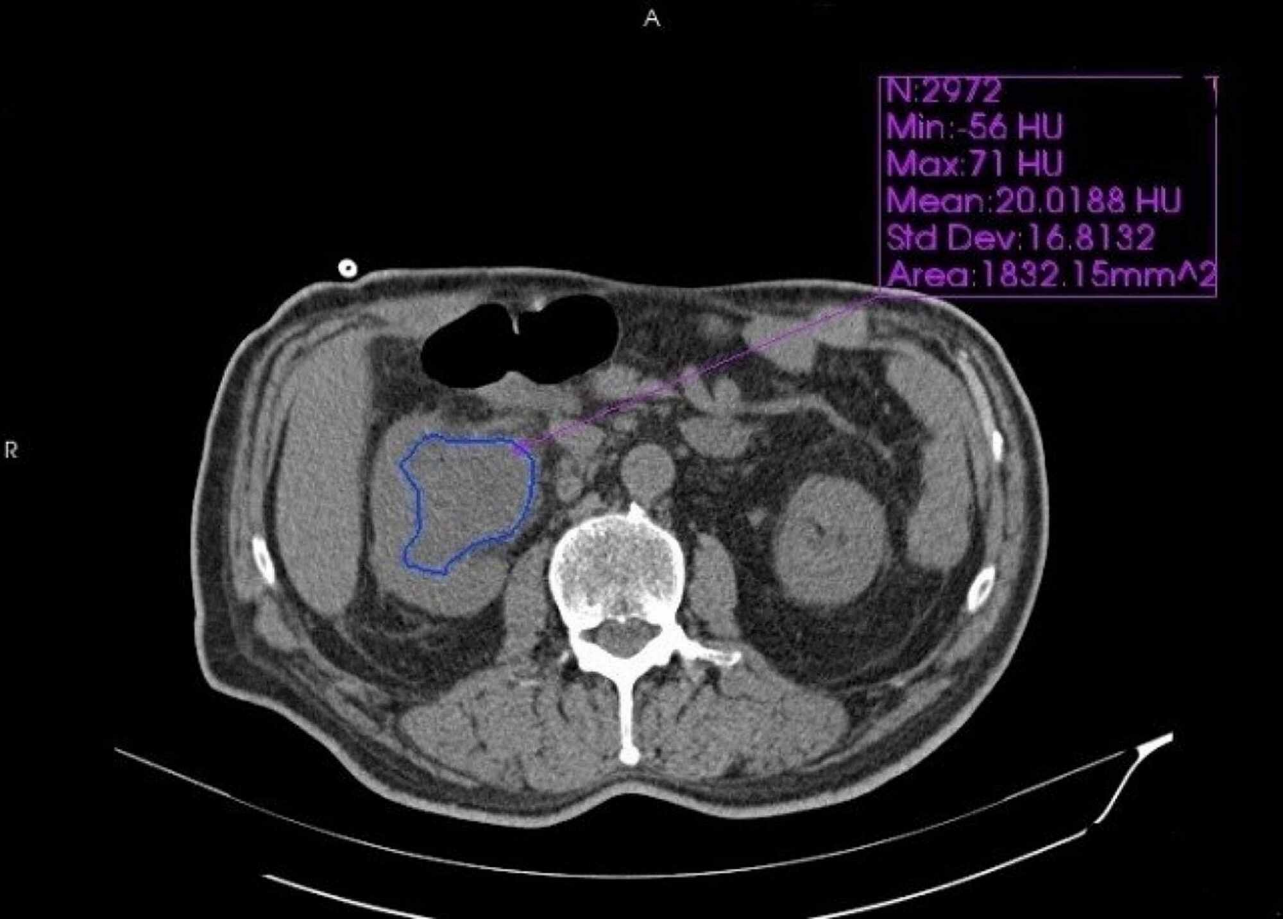
Measured values of fluid in the dilated renal pelvis in a patient diagnosed with pyonephrosis

**Figure 2:**
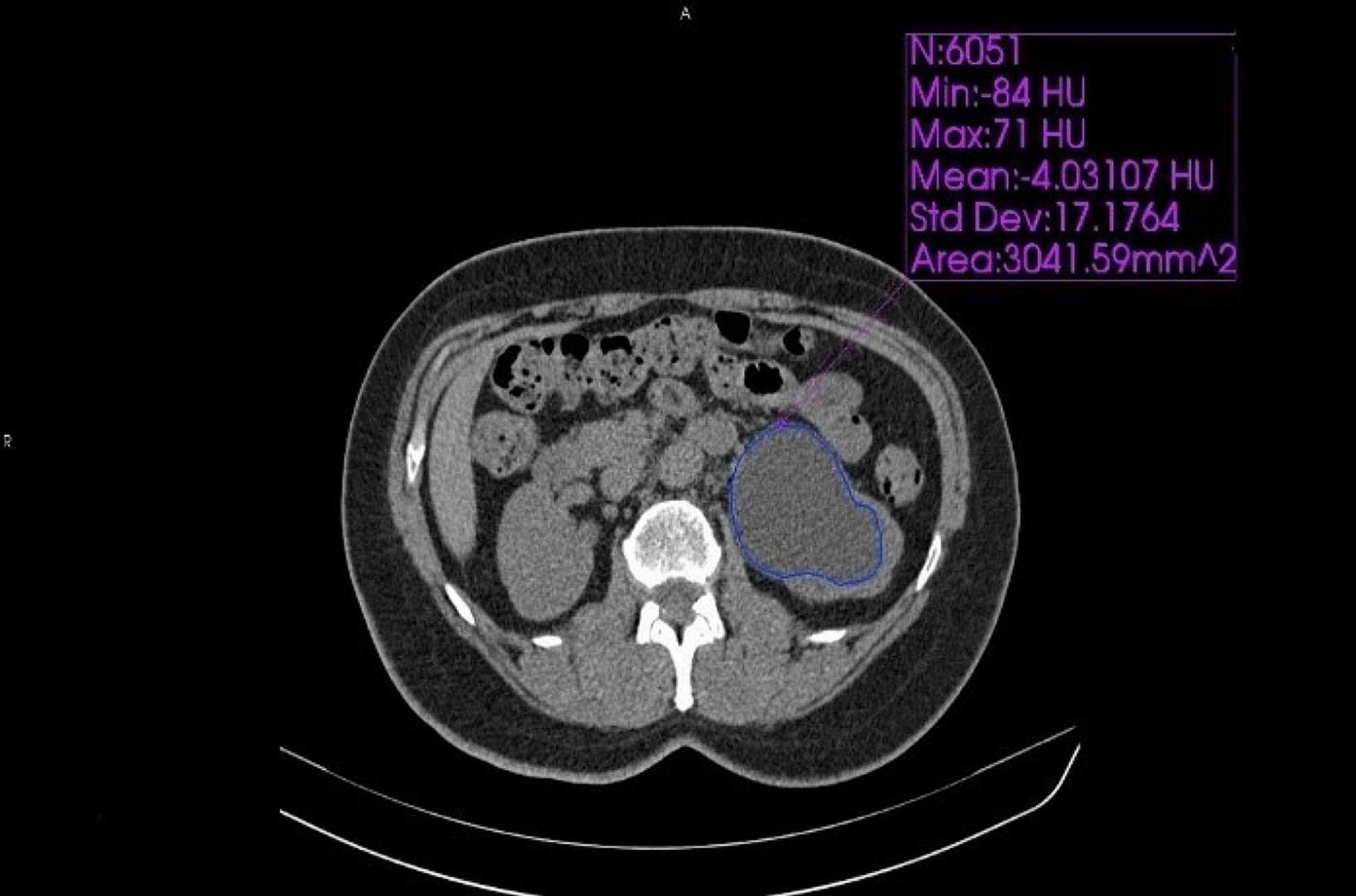
Measured values of fluid in the dilated renal pelvis in a patient diagnosed with hydronephrosis

All CT examinations were performed using a 16-row multi-detector CT device (Somatom Emotion 16, Siemens Healthcare, Erlangen, Germany). The abdominal CT imaging parameters were as follows: collimation of 5 mm, tube current of 150 mAs, field of view of 300 mm, and matrix of 512 x 512. The patients’ renal pelvic anteroposterior (AP) diameter, renal pelvic area, mean HU value, WBC count, creatinine, and glomerular filtration rate (GFR) values were recorded. HU, renal pelvic AP diameter, renal pelvic area, and mean HU value were quantitatively measured by two researchers blinded to clinical data using the free drawing region-of-interest (ROI) method, and the averages of two measurements were taken (Figures 1, 2).

The data were analyzed using SPSS Version 25.0 (IBM Corp., Armonk, NY, USA). The Shapiro-Wilk test was used to evaluate the fit of the data to the normal distribution curve. The data showing normal distribution were presented as mean ± standard deviation (SD). Student’s t-test was used for the comparison of normally distributed data, and the Mann-Whitney U-test was used for the data that did not meet the normality assumption. The categorical data were compared using the chi-square test. The receiver operating characteristic (ROC) curve was utilized to evaluate the efficacy of the HU value in predicting pyonephrosis. A p-value of <0.05 was considered statistically significant.

## Results

A total of 159 patients over the age of 18 years who met the criteria were included in the study. According to the material aspirated at percutaneous needle entry, 123 of the 159 patients were included in the hydronephrosis group and 36 in the pyonephrosis group. The mean age was 62.02 ± 14.58 years for the hydronephrosis group and 56.44 ± 19.08 years for the pyonephrosis group. PN was performed on a total of 214 renal units: 104 unilateral and 55 bilateral. In the pyonephrosis group, among the patients who underwent bilateral nephrostomy, pus was aspirated bilaterally in two and unilaterally in six cases. For these six patients, the kidney from which pus was not aspirated was also included in the hydronephrosis group. Thus, hydronephrosis was detected in 176 renal units and pyonephrosis in 38 renal units. The male/female ratio was 98/25 in the hydronephrosis group and 23/13 in the pyonephrosis group (Table 1). No statistically significant difference was observed between the measured AP diameter and renal pelvic area of the two groups (28.45 ± 10.1 mm vs. 31.13 ± 14.4 mm, p = 0.36 and 658.51 ± 433.1 mm^2^ vs. 755.14 ± 470.6 mm^2^, p = 0.22, respectively). There was also no significant difference between the two groups in terms of the creatinine and GFR values (2.65 ± 1.91 mg/dL vs. 2.20 ± 1.86 mg/dL, p = 0.23 and 50.14 ± 28.21 mL/min/1.73 m^2^ vs. 54.79 ± 38.43 mL/min/1.73 m^2^, p = 0.34, respectively). The WBC values were statistically significantly higher in the pyonephrosis group as expected (9.562 ± 5.264 10^3^/mL vs. 14.699 ± 8.220 10^3^/mL, p = 0.005). When the culture results of the samples obtained from the two groups were examined, 13 (7.38%) of the 176 samples in the hydronephrosis group and 27 (71.05%) of the 38 samples in the pyonephrosis group showed growth, with a statistically significant difference (p < 0.001). In the comparison of the mean HU values between the two groups, the HU value of the pyonephrosis group was significantly higher (2.30 ± 5.02 vs. 10.97 ± 6.68, p < 0.001).

**Table 1:** Comparison of the Demographic Data, Renal Pelvic Area, HU Measurement Values, and Laboratory Results of the Patients in the Hydronephrosis and Pyonephrosis Groups PN, percutaneous nephrostomy; AP, anteroposterior; HU, Hounsfield unit; WBC, white blood cell; GFR, glomerular filtration rate

	Hydronephrosis	Pyonephrosis	p-Value
Number of patients who underwent PN, n	123	36	
Unilateral n (%)	76 (61.8)	28 (77.8)	0.11
Bilateral, n (%)	47 (31.2%)	8 (22.2%) unilateral in six patients; bilateral in two pus aspirated	
Total renal units	176	38	
Gender, n (%)			
Male	98 (79.7)	23 (63.9)	0.74
Female	25 (20.3)	13 (36.1)	
Age, years	62.02 ± 14.58	56.44 ± 19.08	0.25
Etiology, n (%)			
Stone	28 (22.8)	13 (36.1)	0.08
Cancer	79 (64.2)	17 (47.2)	
Stricture	16 (13)	5 (13.9)	
Side, n (%)			
Right	48 (39)	17 (47.2)	0.44
Left	75 (61)	19 (52.8)	
AP diameter, mm	28.45 ± 10.1	31.13 ± 14.4	0.36
Renal pelvic area, mm^2^	658.51 ± 433.1	755.14 ± 470.6	0.22
Mean renal, HU	2.30 ± 5.02	10.97 ± 6.68	<0.001
Renal pelvic urine culture, n (%)			
Positive	13 (7.38)	27 (71.05)	<0.001
Negative	163 (92.61)	11 (28.94)	
WBC, 10^3^/mL	9.562 ± 5.264	14.699 ± 8.220	0.005
Creatinine, mg/dL	2.65 ± 1.91	2.20 ± 1.86	0.23
GFR, (mL/min/1.73 m^2^	50.14 ± 28.21	54.79 ± 38.43	0.34

**Figure 3:**
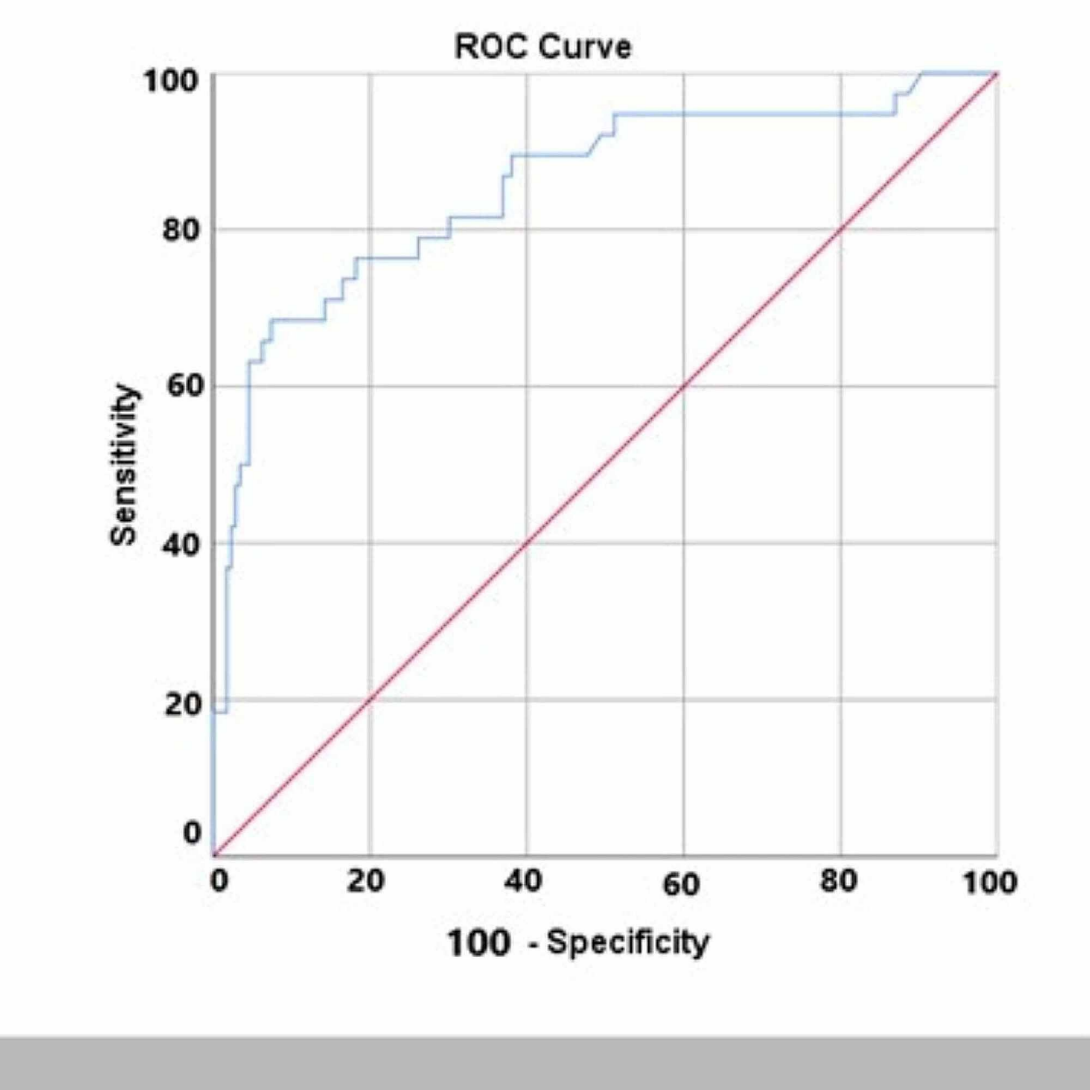
Sensitivity and Specificity Values of the Hounsfield Unit (HU) Measurement for the Differentiation Between Hydronephrosis and Pyonephrosis by ROC Analysis Area under the curve = 0.854 ± 0.038; 95% CI: 0.779-0.929

ROC analysis was performed to investigate the efficacy of the HU value in predicting pyonephrosis. The area under the curve in the ROC analysis was 0.854 ± 0.038 at 95% confidence interval (CI) (0.779-0.929) (Figure 3). The cut-off value for HU was calculated according to the Youden index. At the cut-off value of 8.46, HU had a sensitivity of 68.4% and specificity of 92.6% in the diagnosis of pyonephrosis. The positive and negative predictive values of HU were 66.67% and 93.14%, respectively (Table 2).

**Table 2:** Predictive Values of the HU at the Cut-Off Value of 8.46 HU, Hounsfield unit; PPD, positive predictive value; NPD, negative predictive value

	HU > 8.46 test+	HU < 8.46 test-		
Patient	26	12	38	PPD: 26/39 (66.7%)
Healthy	13	163	176	NPD: 163/175 (93.14%)
Total	39	175	214	

## Discussion

Routine imaging is not necessary for the diagnosis of urinary tract infections; however, imaging methods are used in the presence of a complicated urinary tract infection in immunocompromised patients, diabetics, patients with severe symptoms, and those who are unresponsive to antibiotherapy [8]. In the early stage of pyelonephritis, abnormal findings may not be detected on ultrasonography and contrast-enhanced CT (CECT). It is recommended that the CT scan be performed using contrast enhancement [14]. CECT shows heterogeneous enhancement of the parenchyma, thickening of the pelvic wall, and striated enhancement extending from the renal papillae to the cortex, supporting the diagnosis of pyelonephritis. This appearance has been attributed to contrast condensation due to the decrease in contrast flow in infected tubules.

There are very few studies in the literature on the visualization of complicated urinary tract infections by NCECT, which is easier and cheaper than CECT, involves no risk of allergic reactions, and can be performed independently of renal function values. In addition, when the obstruction is considered to be due to urolithiasis, first, an NCECT scan should be performed to prevent opaque material from masking the stone. The results of our study suggest that it is possible to acquire information about the content of the fluid accumulated in the kidney by HU measurement using a single NCECT scan performed independently of the etiology of obstruction. According to our results, when the measured HU value of the fluid accumulated in the dilated renal pelvis on NCECT is >8.46, the case is pyonephrosis at 68.4% sensitivity and 92.6% specificity.

In cases where the upper urinary tract is obstructed for various reasons, it is necessary to provide drainage by PN, sometimes in emergency and sometimes in elective conditions. Two of the most important parameters determining the urgency of the procedure are the presence of pyonephrosis and ARF. While ARF can be easily diagnosed based on the urea, creatinine, and GFR values, it may not be as easy to make a definitive diagnosis for pyonephrosis. Obstruction and hydronephrosis almost always require urgent treatment in patients with a solitary kidney, but urgent intervention is indicated in patients with both kidneys presenting with unilateral obstruction accompanied by pyonephrosis or uncontrolled pain. Due to the high morbidity and mortality rates in pyonephrosis [15], it is important to make this differentiation in the most accurate and rapid way to initiate appropriate treatment. In a pyelonephritis study conducted in Korea, urinary tract obstruction was found to be an independent risk factor for predicting septic shock (odds ratio = 4.4) [16]. Although fever, flank pain, and elevated WBC support the diagnosis of pyonephrosis in patients with obstruction, non-specific findings on ultrasonography and CT are not always sufficient to differentiate between hydronephrosis and pyonephrosis [7,17].

The HU attenuation value is a unit of measure in which air is taken as -1,000 and water as 0, and the density of the remaining tissues is calculated by comparison to these values [9]. HU can be used for treatment selection and preoperative evaluation of urinary system stones [18,19]. In addition, the use of HU in the differentiation of exudate transudates in body fluids has been reported in the literature [20,21]. In these studies, a significant increase in HU values was found when the fluid progressed from transudate to exudate, suggesting that the higher the density of the fluid content, the higher the HU value measured. This is consistent with the results of our study revealing that the mean HU value of the pus-aspirated group was significantly higher than that of the clear urine-aspirated group (2.30 ± 5.02 vs. 10.97 ± 6.68, p < 0.001).

In a study by Yuruk et al., the HU value was found to be significantly higher in patients with pyonephrosis compared to those with hydronephrosis. When the cut-off value was calculated as HU > 9.21, a diagnosis of pyonephrosis was made with a sensitivity of 65.96% and specificity of 87.93% [13]. Similarly, in our study, the HU value was found to be significantly higher in patients diagnosed with pyonephrosis according to the description in Yoder et al. [22] compared to the hydronephrosis group (2.30 ± 5.02 vs. 10.97 ± 6.68, p < 0.001). At the cut-off value of 8.46, HU had 68.4% sensitivity and 92.6% specificity in the diagnosis of pyonephrosis, similar to the findings of Yuruk et al. However, in that study, the authors used contrast enhancement in the CT of some cases. In contrast, we consider to have obtained a more homogeneous group by including only the patients who underwent NCECT.

Basmaci and Sefik evaluated the renal pelvic fluid HU according to the culture positivity of urine samples obtained by percutaneous needle aspiration and reported that regardless of pyonephrosis status, the HU values were significantly lower in the positive culture group (-8.5 vs. 10, p < 0.001) [23]. However, the authors did not refer to the clarity of the aspirated urine or the presence of pus, and they performed the measurements using an elliptic ROI rather than free ROI that allows covering the entire dilated system. Our findings do not match the data of this previous study. In our study, the urine cultures were positive in 27 (71.05%) patients and negative in 11 (11.9%) patients. We consider that this was due to the antibiotic prophylaxis undertaken before the procedure to prevent any systemic infection that might be caused by pyonephrosis. Since we performed our grouping according to the appearance of aspirated material, we did not include the HU values of patients with and without culture growth in the Results section. However, when we examined these values for the purpose of comparison with the findings of Basmaci and Sefik, we determined that in our study, the HU value of the 40 renal units with positive culture was 8.12 ± 8.21, whereas that of the 174 renal units with negative culture was 2.91 ± 5.38, indicating a statistically significant difference (p = 0.001). This supports an increase in the density of the fluid content due to infection in the dilated obstructed system. In our study, we did not include bladder HU measurements because some of the patients who underwent PN presented with anuria and some others with unilateral obstruction did not have sufficient urine in the bladder for these measurements. In addition, as stated in the Introduction section, the content of urine accumulated in the bladder and kidneys may vary in obstructed systems and infection status [5].

The limitations of our study were the retrospective design and the relatively small number of patients in the pyonephrosis group.

## Conclusions

It is possible to contribute to the differential diagnosis of pyonephrosis and hydronephrosis by performing HU measurements after NCECT, which is an easy, rapid, and non-invasive method to clarify the etiology of the disease. If our data are supported by future prospective randomized studies, simple HU measurements in the presence of hydronephrosis in patients scheduled for surgery for renal calculi can be used to detect a possible pyonephrosis status and allow making the necessary preparations. Furthermore, in addition to flank pain, fever, and elevated WBC, the measured HU value can be considered as a diagnostic criterion for pyonephrosis.
